# Supplementary material for: Identifying priority challenges and solutions for COVID-19 vaccine delivery in low- and middle-income countries: A modified Delphi study
Source: PLOS Glob Public Health. 2022 Sep 8;2(9):e0000844. doi: 10.1371/journal.pgph.0000844 (PMC10021567; doi:10.1371/journal.pgph.0000844)
Supplement: S1 Table — (DOCX) [file pgph.0000844.s001.docx]

**S1 Table**. Sources for identifying Delphi panelists with vaccine delivery and implementation expertise.

| **Category** | **Source** |
| --- | --- |
| **Global Immunization Committees** | Current Members - Strategic Advisory Group of Experts on Immunization (SAGE) at the World Health Organization (WHO) |
|  | Former Members - Strategic Advisory Group of Experts on Immunization (SAGE) at the World Health Organization (WHO) |
|  | Scientific Advisory Committee of the Coalition for Epidemic Preparedness Innovations (CEPI) |
| **Global Health Panels** | WHO Panel Discussion on COVID-19 Related Implementation Research |
| **Immunization Reports** | COVAX: The Pillar of the Access to COVID-19 Tools (ACT) Accelerator Structure and Principles |
