# Supplementary material for: Identifying priority challenges and solutions for COVID-19 vaccine delivery in low- and middle-income countries: A modified Delphi study
Source: PLOS Glob Public Health. 2022 Sep 8;2(9):e0000844. doi: 10.1371/journal.pgph.0000844 (PMC10021567; doi:10.1371/journal.pgph.0000844)
Supplement: S2 Table — (DOCX) [file pgph.0000844.s002.docx]

**S2 Table**. **Identification and ranking of important challenges and solutions for the implementation of COVID-19 vaccination programs in low- and middle-income countries.** Cells highlighted in blue met consensus.

|  | **Round I** | **Round II** | | **Round III** | |
| --- | --- | --- | --- | --- | --- |
|  |  | **N. Respondents** | **N (%) Responding Important¹** | **N. Respondents** | **Grade (%) Agreement on Importance²** |
| **Challenges** | |  |  |  |  |
|  | **Infrastructure, and human and material resources** |  |  |  |  |
|  | Inadequate transport infrastructure including poor road conditions, fuel shortages, poor road security, and absent or poorly maintained equipment. | 56 | 39 (69.6) |  |  |
|  | Inadequate cold chain and storage infrastructure, including insufficient and insecure facilities, unreliable power supply, and absent or poorly maintained equipment. | 56 | 48 (85.7) | 38 | B (86.8) |
|  | Insufficient number of trained non-medical personnel, including logisticians and epidemiologists. | 56 | 32 (57.1) |  |  |
|  | Insufficient number of trained medical personnel to staff vaccination programmes, including to appropriately administer vaccines, screen for contraindications, monitor for post-immunization adverse effects, etc. | 55 | 39 (70.9) |  |  |
|  | Insufficient medical equipment, such as syringes, to safely administer vaccines. | 54 | 36 (66.7) |  |  |
|  | Inadequate infection prevention and control measures, including personal protective equipment (PPE) and infrastructure such as ventilation, throughout the vaccination process and at vaccination sites in particular. | 55 | 35 (63.6) |  |  |
|  | Unreliable and inconsistent use of storage and temperature monitoring equipment to ensure vaccine safety and quality. | 55 | 38 (69.1) |  |  |
|  | Disposal of unused vaccines due to poor management practices, such as mismatched supply and demand, at point of service delivery. | 54 | 28 (51.9) |  |  |
|  | **Planning, processes, and operations** |  |  |  |  |
|  | Inadequate microplanning to operationalize national plans at the local level, including plans to identify target populations, acquire resources, address barriers, and develop work plans. | 56 | 46 (82.1) | 39 | NC (79.5) |
|  | Inadequate population and case data to identify priority groups and hotspots for vaccination, particularly for marginalized populations such as Indigenous communities, refugees, and migrants. | 55 | 34 (61.8) |  |  |
|  | Inequitable distribution of vaccines within a country across different geographical areas, and demographic and socioeconomic groups. | 55 | 42 (76.4) | 39 | NC (76.9) |
|  | Broad and inconsistent criteria to define priority groups such as essential workers, frontline workers, and people with comorbidities. | 55 | 22 (40.0) |  |  |
|  | Competing priorities when deciding policies for which priority groups are vaccinated first, second, third, etc. | 55 | 24 (43.6) |  |  |
|  | Inadequate protocols and information management systems for tracking and monitoring the supply and distribution of vaccines, vaccine coverage, doses administered, and medical equipment. | 55 | 38 (69.1) |  |  |
|  | Inadequate data management systems to enable efficient and equitable vaccine appointment registration. | 55 | 39 (70.9) |  |  |
|  | Unreliable vaccination record system to track individual vaccination status and plan for second dose administration. | 55 | 36 (65.5) |  |  |
|  | Inflexible programmes and communication plans to respond to unstable vaccine supplies and rapidly evolving vaccine safety evidence. | 55 | 32 (58.2) |  |  |
|  | Inadequate pharmacosurveillance systems to identify and respond to events supposedly attributable to vaccination (ESAVIs) and adverse events following immunization (AEFIs). | 56 | 38 (67.9) |  |  |
|  | **Communication, community engagement, and access** |  |  |  |  |
|  | Inadequately tailored community engagement approaches to address the concerns of different target populations such as pregnant people, older adults, religious groups, health care workers, and linguistic groups. | 56 | 46 (82.1) | 39 | NC (74.4) |
|  | Inadequate communication strategies to inform populations of vaccine administration site locations and timings. | 55 | 35 (63.6) |  |  |
|  | Mistrust of vaccine safety and effectiveness among health care workers. | 56 | 40 (71.4) |  |  |
|  | Mistrust of vaccine safety and effectiveness among the general population. | 56 | 39 (69.6) |  |  |
|  | Circulation of vaccine misinformation and disinformation by traditional media and social media sources. | 56 | 39 (69.6) |  |  |
|  | Mistrust in local and global authorities responsible for planning, coordinating, implementing, and monitoring vaccination programmes. | 55 | 31 (56.4) |  |  |
|  | Poor accessibility to vaccination sites for particular populations such as older adults, people with disabilities, and mobile populations such as refugees, displaced persons, or migrant labourers. | 56 | 41 (73.2) |  |  |
|  | **Structural factors** |  |  |  |  |
|  | Competing priorities between governments, vaccine donors and implementing partners at the global level. | 55 | 35 (63.6) |  |  |
|  | Impact of vaccine nationalism on vaccine availability, supply, and brand(s) procured. | 55 | 41 (74.6) |  |  |
|  | Inequitable ability of LMICs to acquire vaccines due to limited independent purchasing power and dependence on COVAX. | 56 | 49 (87.5) | 39 | NC (79.5) |
|  | Inadequate multi-sectoral coordination between government ministries such as health, transport, finance, etc. | 55 | 29 (52.7) |  |  |
|  | Corruption, politicization, and inadequate transparency of vaccine rollout within and among countries. | 56 | 40 (71.4) |  |  |
|  | Insufficient operational funding within countries to create infrastructure and mobilize human resources for vaccine distribution and administration. | 55 | 42 (76.4) | 39 | A (92.3) |
|  | High cost of vaccines to individuals in countries or contexts where they are not provided free of charge. | 51 | 35 (68.6) |  |  |
|  | Absence of national regulatory processes to rapidly approve, import, and/or manufacture new vaccines. | 54 | 31 (57.4) |  |  |
|  | Insufficient health system capacity to simultaneously deliver routine primary care and COVID-19 vaccines at the required scale and speed. | 55 | 46 (83.6) | 39 | A (94.9) |
|  | Inability to efficiently adapt existing childhood immunization programmes for adult populations. | 56 | 36 (64.3) |  |  |
|  | Two-tier health systems that lead to inequitable access to vaccines between public and private providers. | 53 | 28 (52.8) |  |  |
| **Solutions** | |  |  |  |  |
|  | **Infrastructure, and human and material resources** |  |  |  |  |
|  | Encourage multi- and inter-disciplinarity in national vaccine planning and roll out, ensuring the participation of experts in medicine, public health, logistics, and social science, etc. | 54 | 38 (70.4) |  |  |
|  | Deploy mobile units to vaccinate remote or hard-to-reach populations. | 54 | 41 (75.9) | 37 | B (81.1) |
|  | Combine vaccination programmes with existing health or social interventions such as infant immunization and community health workers programmes (i.e., horizontal integration). | 54 | 39 (72.2) |  |  |
|  | Develop a variety of user-friendly vaccine registration systems that include online and on-site registration. | 54 | 32 (59.3) |  |  |
|  | Recruit and train sufficient numbers of medical and non-medical personnel, including medical and nursing students and retired medical professionals. | 55 | 40 (72.7) |  |  |
|  | Provide dedicated training for health care workers focusing on interpersonal communication skills that can facilitate addressing concerns and doubts about the vaccines and the vaccination programmes. | 54 | 42 (77.8) | 39 | A (92.3) |
|  | Diversify vaccine and medical product suppliers to minimize supply chain bottlenecks. | 54 | 29 (53.7) |  |  |
|  | Strengthen cold chain capacity through improved transportation, enhanced storage space, temperature monitoring, etc. | 54 | 45 (83.3) | 39 | A (92.3) |
|  | Develop and strengthen vaccine manufacturing capacity in LMICs, especially related to human resources, equipment, protocols, and reliable access to raw materials. | 53 | 35 (66.0) |  |  |
|  | **Planning, processes, and operations** |  |  |  |  |
|  | Develop a transparent decision-making process involving established and credible entities, such as National Immunization Technical Advisory Groups (NITAGs), non-governmental organizations (NGOs), and watchdogs. | 56 | 40 (71.4) |  |  |
|  | Strengthen participation of WHO and UNICEF at the global, regional, and national levels to provide technical support to local governments in LMICs. | 55 | 36 (65.5) |  |  |
|  | Reduce legal barriers to vaccine technology transfer by waiving intellectual property rights, encouraging patent release and compulsory licensing, and stimulating technology transfer so that LMICs can produce vaccines. | 56 | 38 (67.9) |  |  |
|  | Foster multinational partnerships to promote vaccine research, development, manufacturing, and deployment in LMICs. | 55 | 40 (72.7) |  |  |
|  | Develop flexible plans at the national level in anticipation of multiple scenarios to ensure effective response to changing situations such as supply availability, public perceptions, and the epidemiological situation. | 53 | 40 (75.5) | 39 | U (100.0) |
|  | Develop centralized surveillance systems and digital tools that allow for continuous monitoring and evaluation of key vaccine indicators such as doses distributed, vaccine coverage, adverse events following immunization (AEFIs). | 55 | 44 (80.0) | 38 | A (92.1) |
|  | **Communication, community engagement, and access** |  |  |  |  |
|  | Use empirical data and knowledge of local needs to identify priority groups and implement vaccine rollout. | 54 | 39 (72.2) |  |  |
|  | Prioritize the vaccination of populations involved in the maintenance of essential services such as health care, education, and food industry workers. | 56 | 44 (78.6) | 39 | B (87.2) |
|  | Develop communication strategies through the collaboration of multiple partners (e.g., academia, public health agencies, regulators, media) to counter misinformation, disinformation, and vaccine hesitancy. | 55 | 44 (80.0) | 39 | A (94.9) |
|  | Design strategic, context-sensitive risk communication materials and awareness campaigns tailored to different communities, including campaigns targeted at health care workers. | 55 | 43 (78.2) | 38 | B (89.5) |
|  | Collaborate with local traditional, civil, and religious leaders to address concerns such as vaccine hesitancy. | 55 | 50 (90.9) | 39 | A (94.5) |
|  | Provide official proof of vaccination such as vaccine certificates and vaccine passports. | 56 | 35 (62.5) |  |  |
|  | **Structural factors** |  |  |  |  |
|  | Create a new or reformed global mechanism and binding agreement, with clear accountability, to better regulate and ensure equitable access to, and supply of, vaccines to LMICs regardless of their purchasing power. | 55 | 42 (76.4) | 39 | B (82.1) |
|  | Encourage multi-sectoral coordination and whole-of-government action in national vaccine planning and roll out, securing the participation of relevant ministries such as health, transport, finance, etc. | 54 | 35 (64.8) |  |  |
|  | Secure delayed payment for vaccines procured directly from manufacturers. | 50 | 24 (48.0) |  |  |
|  | Increase donor investments to support vaccine procurement at cost and strengthen immunization programmes in LMICs. | 54 | 38 (70.4) |  |  |
|  | Increase donor investments to support local vaccine manufacturing and supply chains. | 54 | 36 (66.7) |  |  |
|  | Invest in the development of thermostable, single-dose, and needle-free vaccine formulations. | 55 | 36 (65.5) |  |  |
|  | Streamline and harmonize regulatory systems to facilitate efficient importation and local approval of certified vaccines. | 54 | 40 (74.1) |  |  |
| **Note**: LMICs, low- and middle-income countries; NC, no consensus  ¹Important defined as responding "extremely important" or "very important" for specified challenge/solution. ²Grade Unanimous (U) is 100% agreement, A is 90-99% agreement, B is 80-89% agreement, and NC is <80% agreement. | | | | | |
