# Supplementary material for: Identifying priority challenges and solutions for COVID-19 vaccine delivery in low- and middle-income countries: A modified Delphi study
Source: PLOS Glob Public Health. 2022 Sep 8;2(9):e0000844. doi: 10.1371/journal.pgph.0000844 (PMC10021567; doi:10.1371/journal.pgph.0000844)
Supplement: Attachment — Submitted filename: Response to reviewers.docx [file pgph.0000844.s003.docx]

**Response to Reviewer Comments**

**May 26, 2022**

**Journal Requirements**:

**Comment 1.** Please update your Competing Interests statement. If you have no competing interests to declare, please state: “The authors have declared that no competing interests exist.”

**Response 1.** Thank you for clarifying this point. We have updated the Competing Interests statement to say “The authors have declared that no competing interests exist.” on P17; L402.

**Comment 2**. In the online submission form, you indicated that “Due to risk of identification, data cannot be made publicly available. However, specific de-identified dataset versions can be made available upon reasonable request to the study team.”. All PLOS journals now require all data underlying the findings described in their manuscript to be freely available to other researchers, either 1. In a public repository, 2. Within the manuscript itself, or 3. Uploaded as supplementary information.
This policy applies to all data except where public deposition would breach compliance with the protocol approved by your research ethics board. If your data cannot be made publicly available for ethical or legal reasons (e.g., public availability would compromise patient privacy), please explain your reasons by return email and your exemption request will be escalated to the editor for approval. Your exemption request will be handled independently and will not hold up the peer review process, but will need to be resolved should your manuscript be accepted for publication. One of the Editorial team will then be in touch if there are any issues.

**Response 2:** Thank you for this important comment to ensure that the data is freely available to other researchers. We have uploaded the data as a supplementary file in the revised submission titled ‘CVC_data_deidentified.xlsx’. The de-identified dataset includes all data from rounds 1, 2, and 3, apart from the free text responses in round 1 due to risk of identification.

**Comment 3.** Please provide separate figure files in .tif or .eps format only and ensure that all files are under our size limit of 10MB.

For more information about how to convert your figure files please see our guidelines: <https://journals.plos.org/globalpublichealth/s/figures>

**Response 3.** We have re-uploaded Figure 1 as a .tiff format and it is titled ‘Figure_1.tiff’

**Comment 4.** Please review your reference list to ensure that it is complete and correct. If you have cited papers that have been retracted, please include the rationale for doing so in the manuscript text, or remove these references and replace them with relevant current references. Any changes to the reference list should be mentioned in the rebuttal letter that accompanies your revised manuscript. If you need to cite a retracted article, indicate the article’s retracted status in the References list and also include a citation and full reference for the retraction notice.

**Response 4.** Thank you for this comment. We have reviewed the reference list and found it to be complete, correct and without any retracted papers.

**Reviewer #1:**

Dear authors, I enjoyed reading your article and I think its importance lies in the high level of the respondents who were certified to be 'experts' in implementation of vaccine programs and vaccine delivery. The challenges are highly relatable, having had personal and firsthand experience with some of these challenges. The solutions, I think, are also tenable and practicable as some of them have already been deployed in some places and have been shown to work.

No response needed, thank you for your positive comment and critical review.

**Comment 5.** My only reservations were in the recruitment of 'experts' and the response rate, though some of the reasons for these have been well captured in the ‘Limitations’ section of this paper.
However, on a personal note, especially given my experience working in the grassroots in Africa, involving workers in the last mile of the vaccine supply chain as respondents will elucidate more practical and localized challenges and solutions to vaccine delivery and implementation of vaccine programs. This is because they have first hand experience on some of these challenges which, many a time, go unreported to the higher level 'experts'. Also, based on their experience, they may have more local solutions to proffer than the top level 'experts'. This is because, in many cases, they figure out how to navigate some of these challenges on their own without input from or reports to the high level 'experts' for them to take note of.

**Response 5.** Thank you very much for this important comment. We agree that involving “last mile” experts would have been extremely interesting and important in order to contrast their views with those of more “high level” experts. However, this was not feasible due to our recruitment strategy (e.g., self-selected and voluntary) and also was out of scope due to the overall aim of the paper to achieve consensus on limitations at a high-level that are affecting many different countries. Recognizing the importance of this limitation, we have included this explanation in the *Limitations* section of the paper on P15; L360-364.

**Comment 6.** The only other issue I have is with the ‘Results’ section. I felt submerged into this section without any ‘forewarning’ of how the section will be presented. This made me to step back a couple of times. You might consider including a short passage on how the section will be presented (based on the rounds and the challenges and solutions identified).

**Response 6.** Thank you for this suggestion to clarify the format of the Results section. We have added a short introductory paragraph at the beginning of the Results section to introduce how the results are presented – see P8; L191-195.

**Reviewer #2:**

**Comment 7.** Recommendation: It'd be beneficial, to come up with plots, depicting the data visually.

**Response 7.** Thank you for this suggestion. In this manuscript we have followed the standard practice for results reporting in Delphi studies by presenting the consensus findings in a table with the rankings using a final consensus scale. However, to ensure readers can examine findings across time, the supplementary material (Supplemental Table 2) provides a detailed summary with results from all three rounds of the Delphi study.

**Comment 8.** It'd be nice to mention in detail, how are the panelists chosen for this study.

**Response 8.** Thank you for this comment and bringing to our attention that it was not clear how panelists were invited to join the study. We have clarified that panelists were invited to participate through email addresses that were found from scanning of grey and peer-reviewed published literature in the *Panelists & Recruitment* section of the *Methods* P5; L104-105 & L116. Additionally, Supplemental Table 1 provides the specific sources we used for identifying Delphi panelists with vaccine delivery and implementation expertise. We have added an additional reference to this table in the text (P5; L113) and if the Editor and Reviewer think this should be in the main text of the paper, we can add it.

**Comment 9.** Did the authors chose people who have worked in developed, developing countries in supply chain management of the vaccines.

**Response 9.** Thank you for this important question. Yes, the vaccine implementation experts in our Delphi study reported work experience in all World Bank regions and across a variety of content areas including logistics and supply chains. We purposively sought representativeness with respect to geographic distribution and content area expertise, which we have clarified in the *Methods* section on P5; L108. Please see distributions of these demographic characteristics on P9; L198-208 and in Table 2.
